# Effects of Tropical Cyclone (TC) Hellen on the north-westward movement of chlorophyll in the northern Mozambique Channel

**DOI:** 10.1371/journal.pone.0292728

**Published:** 2023-11-02

**Authors:** Hao Shen, Linfei Bai, Haojie Huang, Xiaoqi Ding, Rui Wang, Haibin LÜ

**Affiliations:** 1 Jiangsu Key Laboratory of Marine Bioresources and Environment /Jiangsu Key Laboratory of Marine Biotechnology, Jiangsu Ocean University, Lianyungang, Jiangsu province, China; 2 School of Marine Technology and Geomatics, Jiangsu Ocean University, Lianyungang, Jiangsu province, China; 3 Lianyungang Meteorological Bureau, Lianyungang, Jiangsu province, China; 4 Co-Innovation Center of Jiangsu Marine Bio-industry Technology, Jiangsu Ocean University, Lianyungang, Jiangsu province, China; ULAB: University of Liberal Arts Bangladesh, BANGLADESH

## Abstract

An intense tropical cyclone (TC), TC Hellen, occurred in the northern Mozambique Channel on March 27, 2014, and moved from the east coast of the African continent to the northern Madagascar island. TC Hellen dramatically altered the marine environment in the northern Mozambique Channel, resulting in a significant chlorophyll-a (Chl-a) bloom. A giant surface Chl-a northwest-ward movement from the northwest coast of Madagascar Island was first observed after the passage of TC Hellen in the northern Mozambique Channel. The dynamic mechanisms of these phenomenon were studied by satellite remote sensing, multisource reanalysis data, and Argo float data. The results show that transient northwestward-moving eddies, upwelling, and winds had important effects on the Chl-a bloom and its northwestward movement. Ekman transport driven by coastal southeasterly winds entrained waters with high Chl-a concentrations to the northwest, while TC Hellen enhanced cyclonic eddy upwelling and uplifted nutrient-rich deep water to the upper ocean. This vertical mixing and upwelling in turn triggered the Chl-a bloom in the offshore surface layer. This study provides insight into the reflection of phytoplankton dynamics by TCs in the northern Mozambique Channel.

## Introduction

A tropical cyclone (TC) is an atmospheric cyclonic structure with a warm center that usually occurs over low-latitude oceans. As a TC occurs, it gradually develops into a tropical storm (TS), usually accompanied by strong winds, heavy rainfall, and storm surges that can cause severe damage [1–3]. As the ionosphere is disturbed during the passage of the TC, the positioning accuracy of GNSS is reduced [4, 5]. TCs bring strong winds and high waves that can cause vibration and displacement of submarine pipelines, resulting in damage [6]. Furthermore, near-inertial internal waves have also been observed during and after TC passage, which can exert complex force and torque on cylindrical tendon legs [7]. TCs are not very frequent off the southeast coast of the African continent compared to the northwest Pacific and North Atlantic regions. However, TCs can make landfall and cause storm surge damage to coastal infrastructure and marine ecosystems [8]. Therefore, analyses of the marine and atmospheric environment are required to predict the trajectories of TCs in this region [9]. Generally, surface Chl-a concentrations can increase during and after the passage of TCs [10]. However, not all TC events can trigger phytoplankton blooms [11, 12]. This mainly depends on whether the ocean dynamic process triggered by TCs can increase the supply of nutrients [12].

Previous studies have shown several complex physical processes associated with Chl-a blooms, including TC internal dynamics, the surrounding flow field, sea-air interactions, and the TC intensity. The oceanic mixed layer in the upper layer has the same density and temperature [13, 14]. The three main processes caused by TC included turbulent mixing, land-based runoff enhancement, and resuspension, which can increased nutrient concentrations in seawater, resulting in improved primary productivity [15, 16]. TCs can cause enhanced runoff and a large influx of nutrients into offshore waters [17, 18]. Enhanced upwelling can lead to increased nutrient availability in the photosynthetic layer [19–21]. Li et al. proposed three types of typhoon-induced upwelling that enhance coastal algal blooms [21]. Air-sea interactions play an essential role in TC development [22] when energy and water vapor are transported through sensible heat and latent heat exchanges at the sea-air interface [23]. The vertical uplift of Chl-a and nitrate by upwelling in cyclonic eddies can increase outbreaks of surface Chl-a [24, 25]. It is important to research TC development based on the upper ocean’s characteristics and TC feedback to ocean eddies [26–28]. The reaction of the upper ocean to TCs depends on the TC intensity, traveling speed, and oceanic environment, and these factors can be manifested in various oceanic parameters, such as the sea surface temperature (SST), ocean stratification, ocean currents, and chlorophyll concentration [29, 30]. As TCs pass through the upper ocean, surface currents can form a clockwise cyclonic eddy in the Southern Hemisphere, which can lead to a thickening and deepening of the mixed layer and a weakening and thinning of the stratification. At the same time, strong winds and large waves generate the upwelling and cold bottom water upwelling, resulting in a decrease in SST and a subsequent increase in nutrients. These processes can lead to chlorophyll blooms in the upper ocean.

In this paper, we study the dynamic mechanisms of the Chl-a bloom in Box A and the Chl-a northwestward movement from nearshore to offshore of northern Madagascar Island along the track of TC Hellen. Interestingly, a northwestward movement of Chl-a from the northwest coast of Madagascar Island was first observed after the passage of TC Hellen. This study hopes to provide new insights into the ecodynamic response of phytoplankton to TC processes in the northern Mozambique Channel.

## Data and methods

### Study area

**Fig 1:**
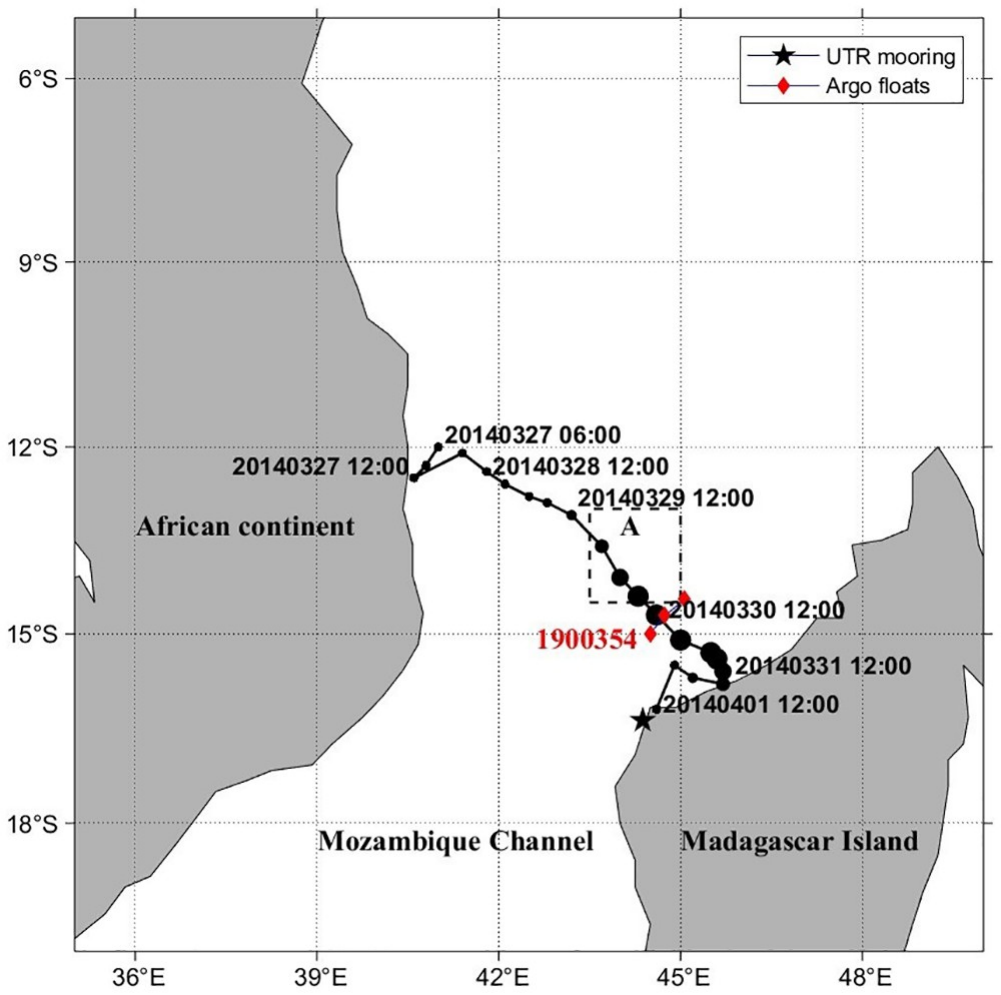
Map of the study area showing the track of TC Hellen (March 27–April 1, 2014) in the Mozambique Channel. The locations of the TC center are indicated by circles in the time format of year-month-date-hour. Diamonds mark the positions of the Argo floats (1900354). The pentagram marks the parts of the UTR mooring.

The study area is located in the northern Mozambique Channel, southeast of the African continent. The Mozambique Channel is a north-south strait between the African continent and Madagascar Island [31, 32]. The unique oceanographic shape and location of the region favor the formation of mesoscale eddies that move and spread southward [33], making it one of the most complex and active regions for oceanic eddies [34, 35]. Ocean eddies can play an important role in the development of TCs [14, 36]. TC Hellen formed in the northern Mozambique Channel on March 27, 2014, and moved southeast. During this period, the slowest travelling speed of the TC was 0.07 m/s (Table 1). Finally, it made landfall on the northwest coast of Madagascar on April 1. Surface Chl-a concentrations continued to increase from April 1 to April 5 in Box A (14.5°S-13°S, 43.5°E-45°E), where a cyclonic eddy developed during the passage of TC Hellen (Fig 1).

**Table 1:** Position, time, intensity, travelling speed and minimum sea level pressure of TC Hellen.

Latitude	Longitude	Time	Intensity (kts)	Travelling speed(m/s)	Minimum Sea Level Pressure (mb)
120S	410E	3/27/2014 06	20		1007
123S	408E	3/27/2014 12	25	1.85	1004
125S	406E	3/27/2014 18	25	1.45	1004
122S	410E	3/28/2014 00	30	2.57	1000
121S	414E	3/28/2014 06	30	2.12	1000
124S	418E	3/28/2014 12	30	2.57	1000
126S	421E	3/28/2014 18	35	1.85	996
128S	425E	3/29/2014 00	40	2.30	993
129S	428E	3/29/2014 06	45	1.63	989
131S	432E	3/29/2014 12	55	3.17	982
136S	437E	3/29/2014 18	65	3.63	974
141S	440E	3/30/2014 00	90	3.00	956
144S	443E	3/30/2014 06	115	2.18	937
147S	446E	3/30/2014 12	130	2.18	926
151S	450E	3/30/2014 18	130	2.91	926
153S	455E	3/31/2014 00	125	2.77	929
154S	456E	3/31/2014 06	100	0.07	948
156S	457E	3/31/2014 12	75	1.15	967
158S	457E	3/31/2014 18	60	1.03	978
157S	452E	4/01/2014 00	40	2.62	993
155S	449E	4/01/2014 06	30	3.74	1000
162S	446E	4/01/2014 12	25	3.91	1004

Note: Times are given in UTC.

### Data

The Joint Typhoon Warning Center (JTWC) presents the track data for “Hellen,” accessed via https://www.metoc.navy.mil/jtwc/jtwc.html; this dataset contains the time, maximum wind speed, and location of the TC center within 6 hours.

The daily Chl-a concentration was presented from the GlobColor project, which provides a continuous portfolio of L3 ocean color products with a spatial resolution of 4 km (http://hermes.acri.fr/index.php?class=archive). Daily microwave and infrared interpolated sea surface temperatures (SSTs) with a spatial resolution of 9 km were provided by a remote sensing system (https://data.remss.com/).

The seawater current velocity, seawater temperature, salinity datasets, gridded sea level anomaly (SLA) data at a resolution of (1/12)° × (1/12)° and subsurface daily Chl-a concentration dataset at a resolution of (0.25)° × (0.25)° are obtained from the Global Marine Copernicus Marine Environmental Monitoring Service (CMEMS) merged grid product (https://resources.marine.copernicus.eu/; https://doi.org/10.48670/moi-00021 and https://doi.org/10.48670/moi-00019).

Latent heat fluxes with resolutions of (0.25)° are available from the NOAA Climate Data Record (CDR) of Ocean Heat Fluxes, version 2 (https://www.ncei.noaa.gov/).

The wind vector data at 10 m above the sea surface are from RSS, Cross-Calibrated Multi-Platform (CCMP, http://www.remss.com/measurements/ccmp), which has a spatial resolution of (0.25)° × (0.25)°.

The Argo program provided the vertical profile data for temperature, salinity, and Brunt–Vaisala frequency (http://www.argodatamgt.org/). Argo buoy data with station number 1900354 were used in this study.

### Methods

#### Ekman pumping velocity (EPV)

The vertical mixing in the upper ocean can be effectively researched based on Ekman pumping [37, 38]. The EPV can cause the convergence and divergence of seawater [39] and can be expressed as follows:

$$EPV=curl\;\left(\frac{\Delta \times \tau }{\rho_{f}}\right)$$ (1)

$$\tau =\rho_{a}C_{d}u^{2}$$ (2)

f=2Ωsinθ (3)

where *f* is the Coriolis parameter, Ω is the rotational angular velocity of the Earth, *θ* is the latitude, the curl of surface wind stress is represented by Δ × *τ*, *ρ*_a_ is the density of air, *ρ* is the density of seawater and u is the wind speed at 10 m above the sea surface.

#### Mixed Layer Depth (MLD) and Interface Layer Depth (ILD)

MLD is the depth at which the potential density increase relative to the sea surface is equal to the potential density increase at the sea surface caused by a 0.5°C decrease in sea surface temperature (SST). ILD is the depth at which the temperature is 0.5 °C lower than that of the surface (10 m) [40].

$$\Delta \sigma_{\theta }=\sigma_{MLD}-\sigma_{10}$$ (4)

$$\Delta \sigma_{\theta }=\sigma_{\theta }(T_{10}+\Delta T,S_{10},P_{0})-\sigma_{\theta }(T_{10},S_{10},P_{0})$$ (5)

$$\text{MLD}=D(\sigma_{MLD})$$ (6)

where Δ*σ*_*θ*_ is the potential density increase relative to the surface, Δ*T* = -0.5℃, *T*_10_ and *S*_10_ are the temperature and salinity values at the reference surface (10 m), and *P*_0_ is the pressure at the ocean surface.


$$\Delta T=T_{ILD}-T_{10}$$ (7)

$$\text{ILD}=D(T_{ILD})$$ (8)

#### Brunt–Vaisala frequency

The Brunt-Vaisala frequency can be used to represent the vertical stability of seawater [41] and can be expressed as follows:

$$N=\sqrt{\frac{-g}{\rho }\times \frac{d\left(\rho \right)}{d\left(z\right)}}$$ (9)

where *ρ* is the potential density, **g** is the gravitational acceleration, and *z* is the geometric height. The depth of the thermocline can be determined from the maximum Brunt–Vaisala frequency [38].

#### Tropical cyclone heat potential (TCHP)

The 26 °C isotherm depth (D_26_) is the depth in the ocean where the water temperature is at or above 26 °C and can be used to reflect the ocean heat content (OHC) of the upper ocean [42]. TC formation requires SST over 26 °C [43]. TCHP is an essential oceanic parameter for studying cyclones and hurricanes [44].

$$TCHP=\rho C_{P}\int_{0}^{D_{26}}\left(T-26\right)\;dz$$ (10)

where *ρ* is the density of the seawater, C_P_ is the specific heat capacity of seawater, T is the water temperature at dz, and D_26_ is the depth of the 26 °C isotherm.

#### Statistics

We performed statistical analyses on temporal data sets in Box A (Figs 4, 6, and Table 2). We calculated the arithmetic mean of the time series in Box A by plotting chlorophyll concentration, SST, latent heat flux, and TCHP for data analysis.

**Table 2:** Comparison of latent heat flux, TCHP, SST, and MWS data in Box A during the passage of TC.

	latent heat flux(W/m^2^)	TCHP(kJ/cm^2^)	SST(℃)	MWS(m/s)
Before TC adoption	137.99	74.72	29.5	60.00
After TC adoption	70.05	55.01	27.9	16.30
Net loss	67.94	19.71	1.6	43.70

## Results

### Surface Chl-a

**Fig 2:**
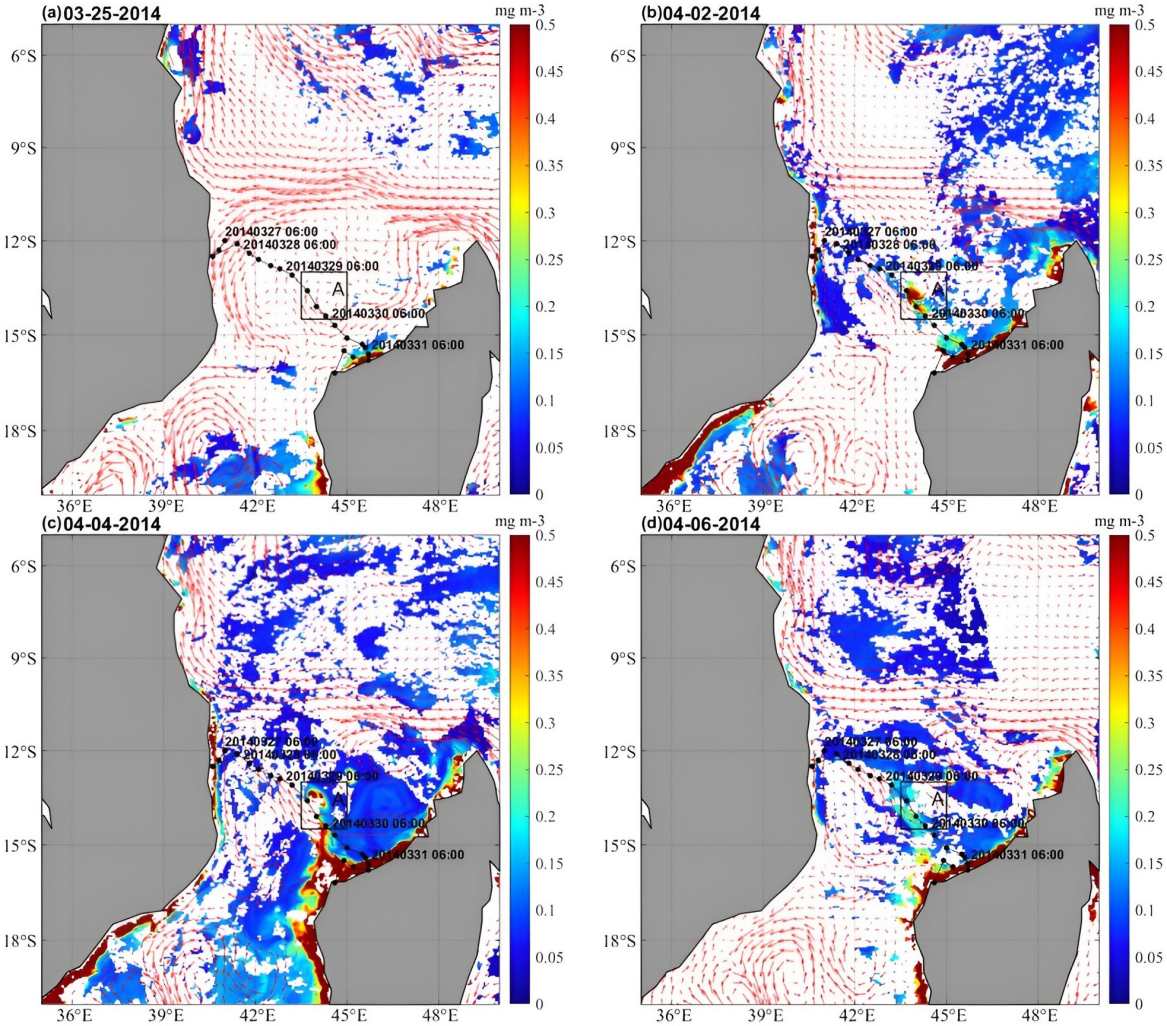
Sea currents and MODIS Chl-a concentrations (mg∙m^-3^) showing the evolution of the Chl-a event between March 25 and April 6, 2014: (a) March 25, (b) April 2, (c) April 4, and (d) April 6, 2014. (Note: The daily Chl-a concentration data is from GlobColor project; http://hermes.acri.fr/index.php?class=archive. Sea currents data are from E.U. Copernicus Marine Service; https://doi.org/10.48670/moi-00021).

**Fig 3:**
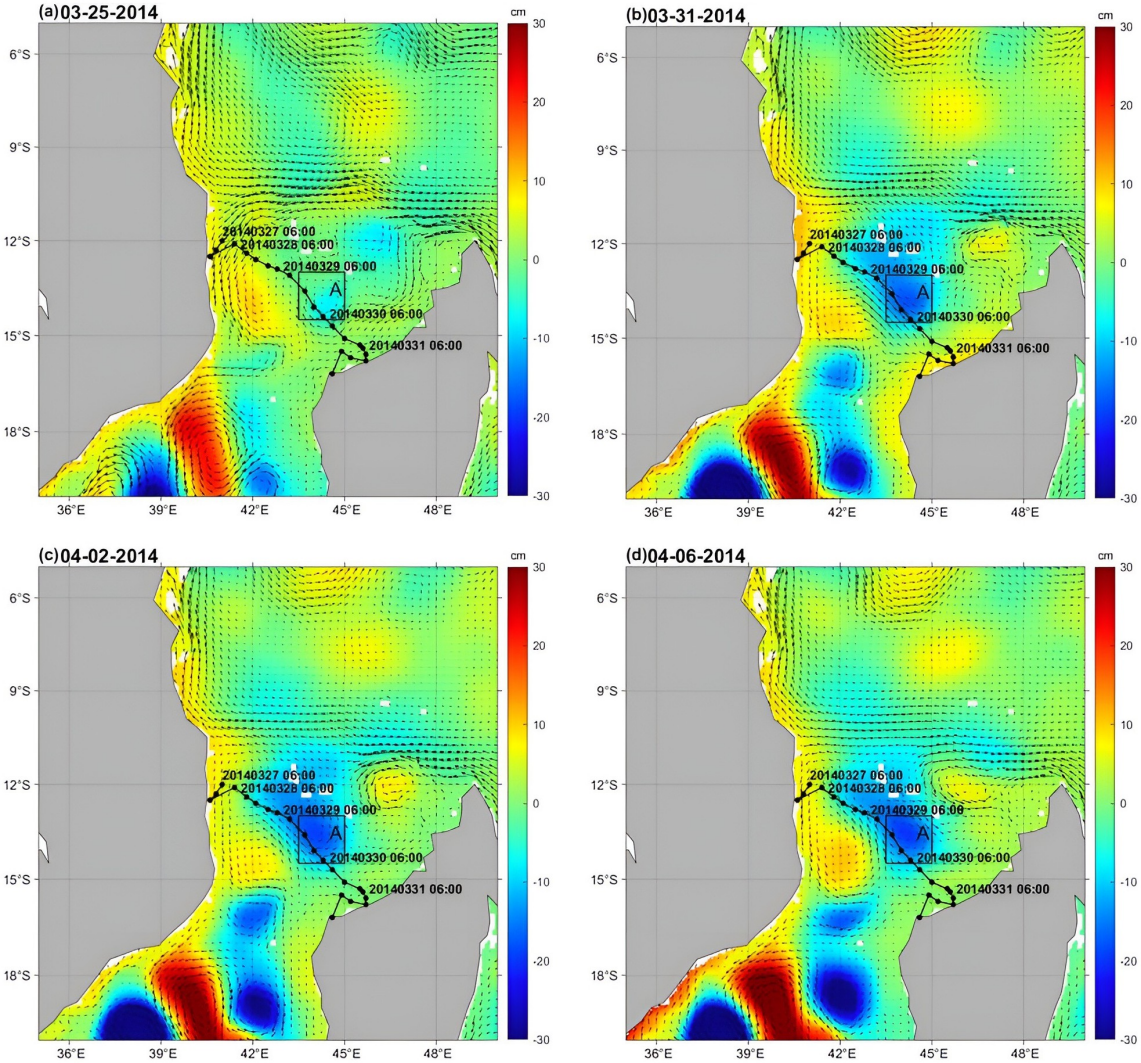
Snapshots of SLA (cm) and sea currents on (a) March 25, 2014, (b) March 31, 2014, (c) April 2, 2014, and (d) April 6, 2014. (Note: SLA and sea currents data are from E.U. Copernicus Marine Service; https://doi.org/10.48670/moi-00021).

Fig 2 shows the evolution of the Chl-a concentration before and after the passage of TC Hellen. Interestingly, Chl-a on the northwest coast of the island of Madagascar experienced a northwestward movement after the passage of TC Hellen (Fig 2a–2c). The surface Chl-a bloom occurred along the track of the TC between March 25 and April 6, moving northwestward from nearshore to offshore. On April 2 (Fig 2b), a Chl-a bloom (>0.4 mg∙m^-3^) was observed in Box A, and the coastal Chl-a concentrations were higher than those before the TC passage. On April 4, the Chl-a concentration was greater than 0.4 mg∙m^-3^, and Chl-a moved northwestward from the coast of northern Madagascar Island (Fig 2c). On April 6, Chl-a spread northwestward to the middle of the strait with submesoscale filaments (Fig 2d). B.S. Malauene et al. (2014) found a similar phenomenon in the northern Mozambique Channel, where high Chl-a concentrations moved southeastward along the African coastline to the middle of the Mozambique Channel due to the influence of the distinctive eddies in the Mozambique Channel [32]. After TC Hellen passed through the Mozambique Channel, causing two eddies of opposite polarity (Fig 3). We first found a northwestward movement of chlorophyll along the fronts of the two eddies. The possible dynamic mechanisms of the Chl-a bloom in Box A are analyzed in the next section.

### SLA and sea currents

The ground-rotation velocity (unit: cm∙s^-1^) and SLA (unit: cm) from March 25 to April 6 are shown in Fig 3. On March 25, a cyclonic (cold) eddy near Box A at 43.5°~ 45°E and 13°~ 14.5°S was relatively weak before the passage of Hellen (Fig 3a), with an SLA of -10 cm and an eddy diameter of about 160 km. On March 31, the SLA in Box A appeared at approximately -20 cm during the passage of the TC (Fig 3b). With the passage of TC Hellen, the eddy in Box A gradually increased in the diameter to 275 km, and a strong northwestward current occurred at the fronts between the northern cyclonic (cold) eddy and the southern anticyclonic (warm) eddy (Fig 3c and 3d). A coastal current along the African continent’s east coast flowed southward from the periphery of the anticyclonic eddy, strengthening the anticyclonic eddy [14]. The two eddies of opposite polarity produced a current dipole pair moving from nearshore to offshore (Fig 3b–3d) [32].

### Sea surface Chl-a and SST in Box A

**Fig 4:**
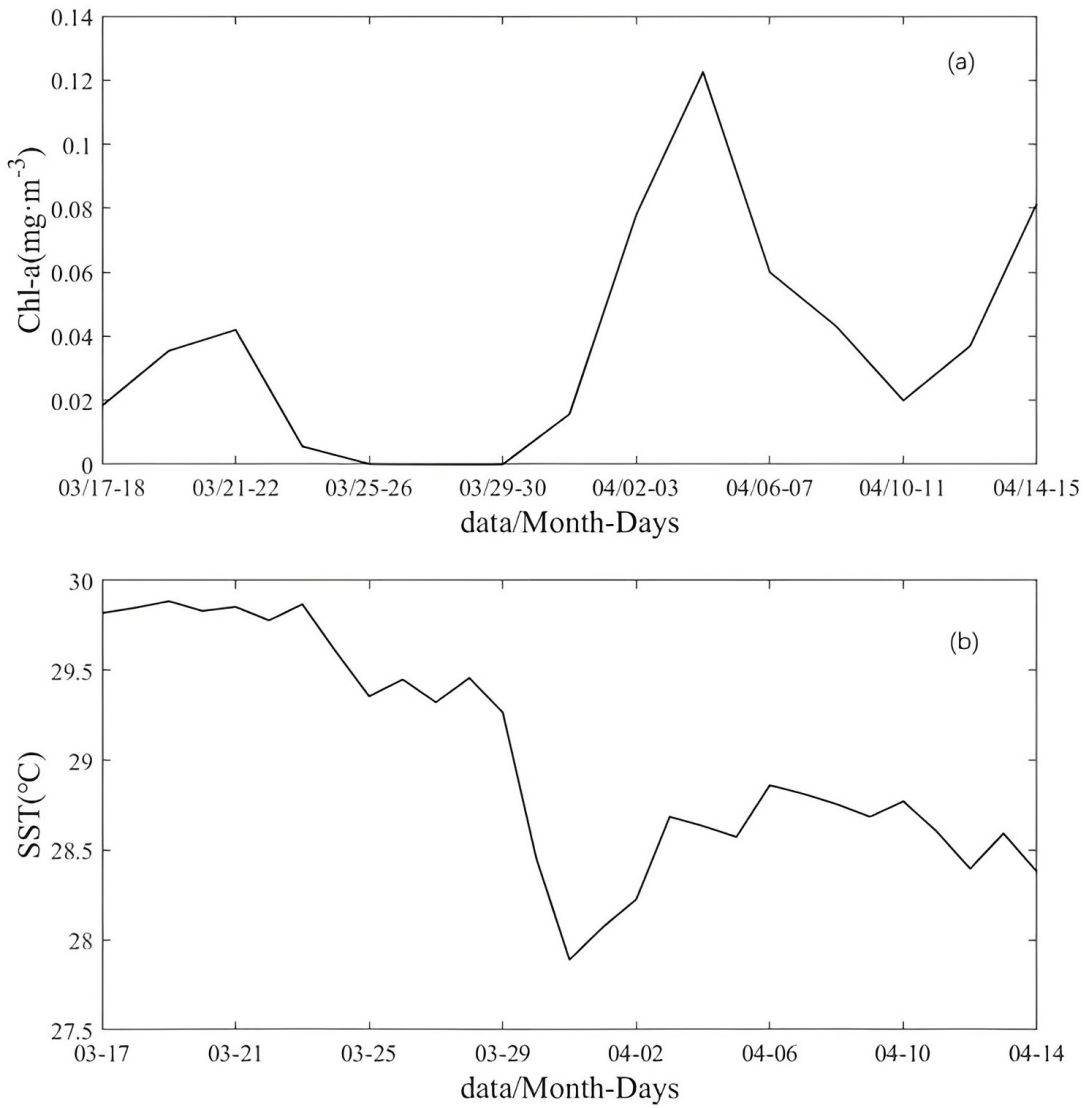
Time series of the spatially averaged SST and time series of the two-day-averaged Chl-a in Box A from March 17 to April 15.

Time series of the averaged surface Chl-a concentration and SST in Box A are shown in Fig 4. To overcome the limited coverage of satellites due to cloud cover, Xia et al. used 2-day averaged observations to show the enhancement of Chl-a [45]. The 2-day averaged Chl-a concentration in Box A is shown in Fig 4a. The Chl-a concentration increased on March 30, reaching a maximum of 0.12 mg∙m^-3^ on April 5, and decreased sharply to 0.02 mg∙m^-3^ after April 10. The Chl-a bloom occurred approximately three days after the passage of the TC and persisted for five days (Fig 4a).

SST cooling is another essential response of the ocean to TCs and can be influenced by the TC intensity and traveling speed [10]. Between March 27 and March 31, the SST in northern Mozambique decreased significantly from 29.5 °C to 27.9 °C (>1 °C) after the passage of the TC (Fig 4b). This was caused by upwelling during and after the passage of the TC, as upwelling can uplift nutrients to the upper ocean.

#### Heat transport and heat potential

**Fig 5:**
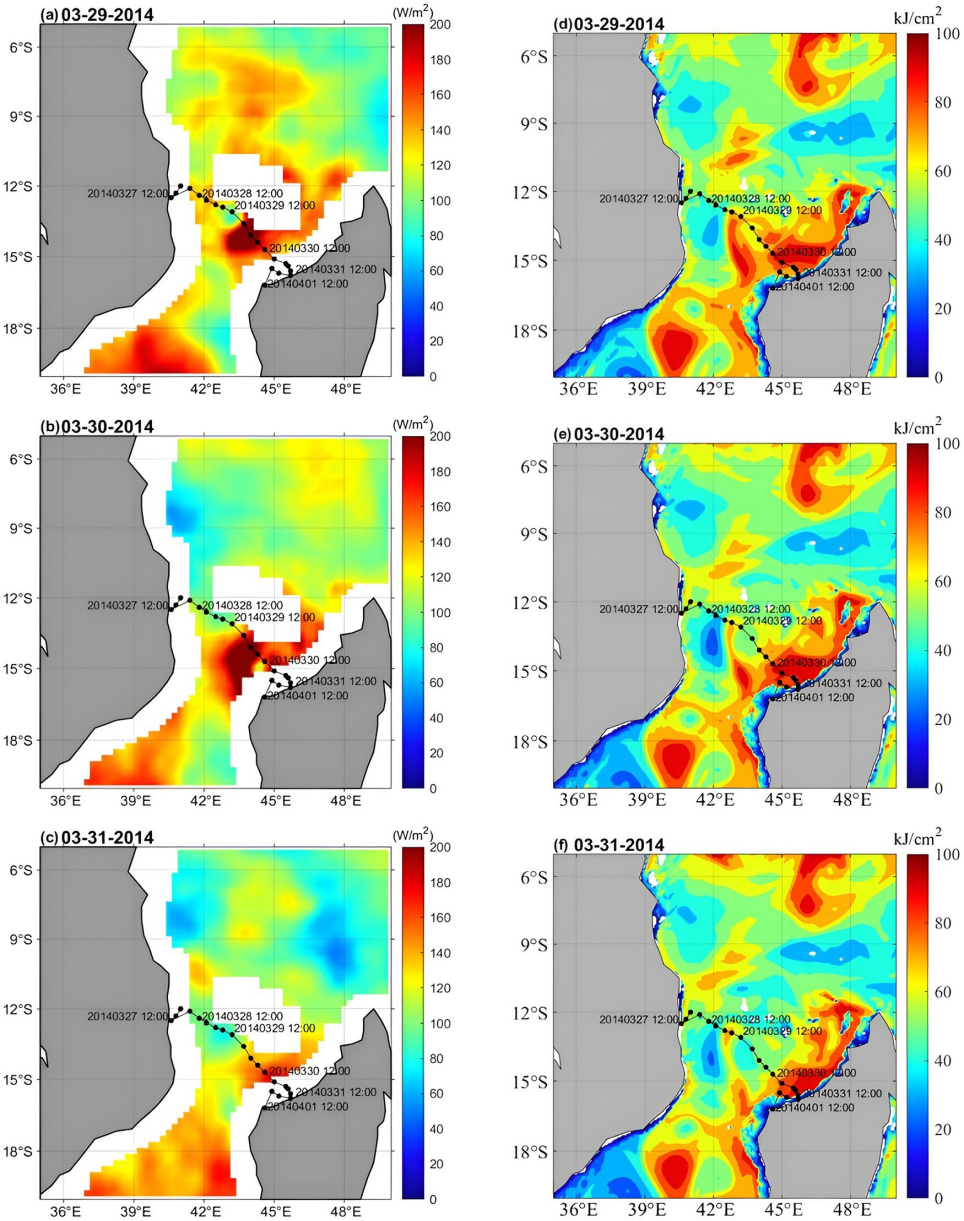
The ocean heat environment during the passage of the TC. Panels (a–c) show the variations in latent heat flux from 29 to March 31, 2014; panels (d–f) show the changes in TCHP from 29 to March 31, 2014.

**Fig 6:**
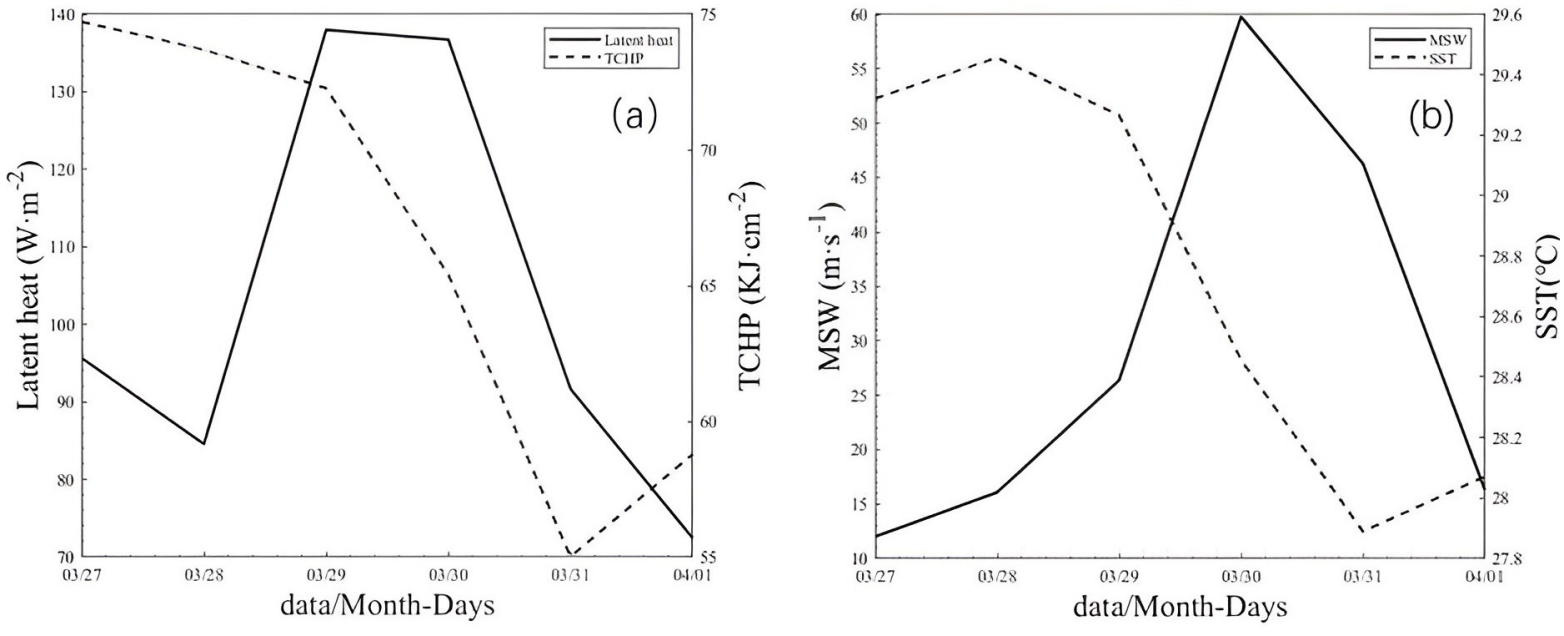
(a) Time series of mean latent heat fluxes and TCHP in the cyclonic eddy and SST from March 27 to April 1, 2014. The solid line and dashed line represent the latent heat fluxes and TCHP, respectively. (b) Time series of mean SSTs in the cyclonic eddy in Box A and the maximum wind speed of the TC from March 27 to April 1. The solid line and dashed line indicate the maximum wind speed and the SST, respectively.

Table 2 provides a comparison of latent heat flux, TCHP, SST, and MWS data in Box A during the passage of TC Hellen. On March 29, the maximum mean latent heat flux above the cold eddy in Box A was 137.99 W/m^2^ (Fig 5a). The cold eddy was located along the TC track and had a strong influence on the TC (Figs 5a–5c and 6a). During the passage of the TC, both the TCHP and oceanic heat content were high in the Mozambique Channel, while the heat content of the cold eddy was significantly lower than that of the warm eddy. The passage of a TC can result in significant heat loss from the upper ocean [46, 47]. The TCHP of the cyclonic (cold) eddy in Box A decreased from 74.72 kJ/cm^2^ on March 27 to 55.01 kJ/cm^2^ on March 31, a loss of 19.71 kJ/cm^2^ (Figs 5d–5f and 6a). After the TC passed over the ocean cold eddy in Box A, the cold eddy limited further TC development. The daily mean maximum wind speed weakened from 60.00 m/s on March 30 to 16.30 m/s on April 1, a significant reduction of 43.70 m/s. Meanwhile, the TC stimulated dramatic SST cooling and a substantial decrease in TCHP. The changes in SST in Box A also coincided with the changes in TCHP, with sustained energy transfer to a maximum on March 30 (Fig 6b). After March 30, the center of the TC moved away from the eddy, the heat loss from the eddy slowed down, and the minimum sea level pressure decreased sharply (Table 1). Walker et al. studied a significant decrease in SST when a TC passed over a cold oceanic eddy [48]. As the TC Hellen passed north of the Mozambique Channel, a cyclonic eddy current field was generated at the sea surface. A cold eddy gradually developed and expanded in the vicinity of Box A (Fig 3). The upwelling induced by the cyclonic eddy in Box A facilitated the transport of cold water from the deep layer to the upper ocean with Ekman pumping. Sea surface cooling could no longer offer heat from the ocean, thereby weakening the TC intensity [44, 49, 50].

## Discussion

### Mixing of ocean eddies

**Fig 7:**
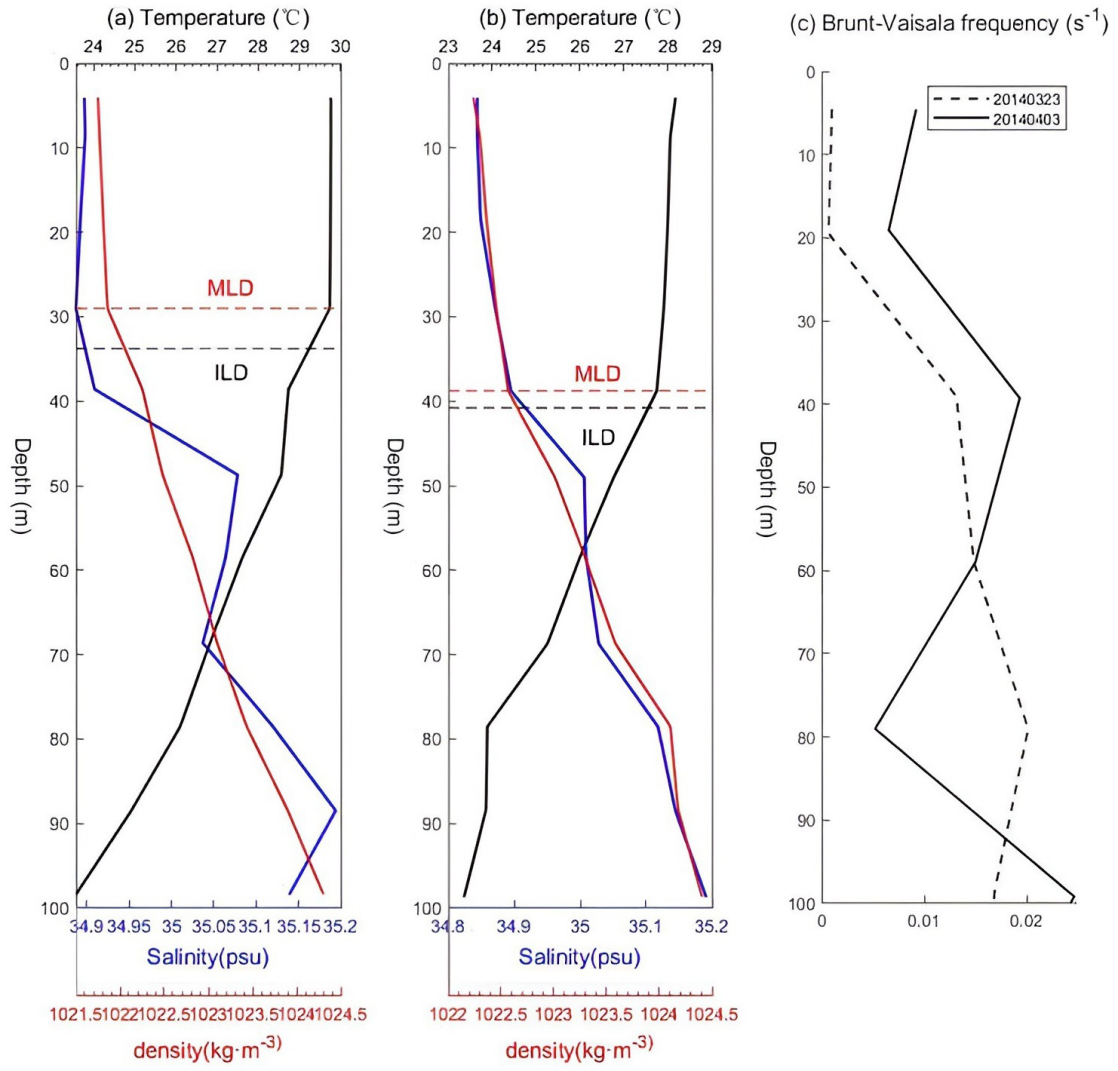
The temperature (black), salinity (blue), and density (red) profiles in panels (a) and (b) were measured by Argo floats 1,900,354 on Mar. 23 and Apr. 03, respectively. The dashed red lines denote the MLD, and the dashed black lines indicate the ILD. (c) Brunt-Vaisala frequencies on Mar. 23 and Apr. 03.

The mixed layer depth (MLD) and isothermal depth (ILD) calculated from Argo data according to the equations provided by He et al. (2020) are shown in Fig 7 [51]. During and after the passage period of TC Hellen, the MLD deepened from 29.03 m to 38.75 m, and the ILD changed from 33.79 m to 40.32 m. As the MLD and ILD deepened, the barrier thickness decreased by 3.2 m (Fig 7). Ocean eddies can provide oceanic environment conditions to enhance and weaken TCs [14]. The latent heat flux from the cyclonic eddies is stronger during the passage of a TC than after the passage of the TC (Fig 6a), while the TCHP decreases dramatically. The strong shear of the TC caused entrainment of the mixed layer and led to sea surface cooling, which caused a reduction in the latent heat flux from the ocean to atmosphere by 64.4 W/m^2^ from 30 March to April 1, weakening the strength of the TC and creating a negative feedback effect.

During the development of the TC, significant thermal changes occurred in the upper ocean, especially over the oceanic eddies [14]. Previous studies on the interaction of mesoscale eddies with TCs have often focused on the enhancement of TCs by warm eddies. Lin et al. suggested that warm eddies can act as effective insulators between typhoons and deep-sea cold water [36]. Lin II found that the presence of warm ocean eddy can effectively isolate the cold, nutrient-rich water to be entrained to the surface ocean [11]. Richard M. Yablonsky and Isaac Ginis proposed that a warm ocean eddy (WCR) has a higher heat content than its surroundings and is generally more conducive to hurricane intensification when a storm core encounters a WCR [52]. In contrast, the responses of cold eddies to transiting TCs have been less reported [53]. Cold eddies have relatively stable thermodynamic structures, which can impact the surface cold zone, the stratification structure, and the intensity of passing TCs [54–56].

Heat loss accompanied by a drop in temperature created a temperature gradient at the sea surface, allowing oxygen and other gases to diffuse from the ocean floor to the surface. This process, known as mixed-layer enhancement, helps to hold gases so that cold water can inhibit the development of TC. Chowdhury et al. (2020) proposed that part of the upper ocean cooling is due to wind-driven evaporative cooling and upwelling of cold water from the subsurface into the upper ocean caused by wind stress of TCs [57]. The strong cold cyclonic eddy (Fig 3d) significantly enhanced SST cooling, uplifting cold deep water to the upper layer, which also suppressed the development of the TC [58, 59]. These are consistent with the results that the weakening effect of the TC was caused by the cold eddy via Ekman pumping, which was triggered by TC Helen.

### Upwelling by Ekman pumping

**Fig 8:**
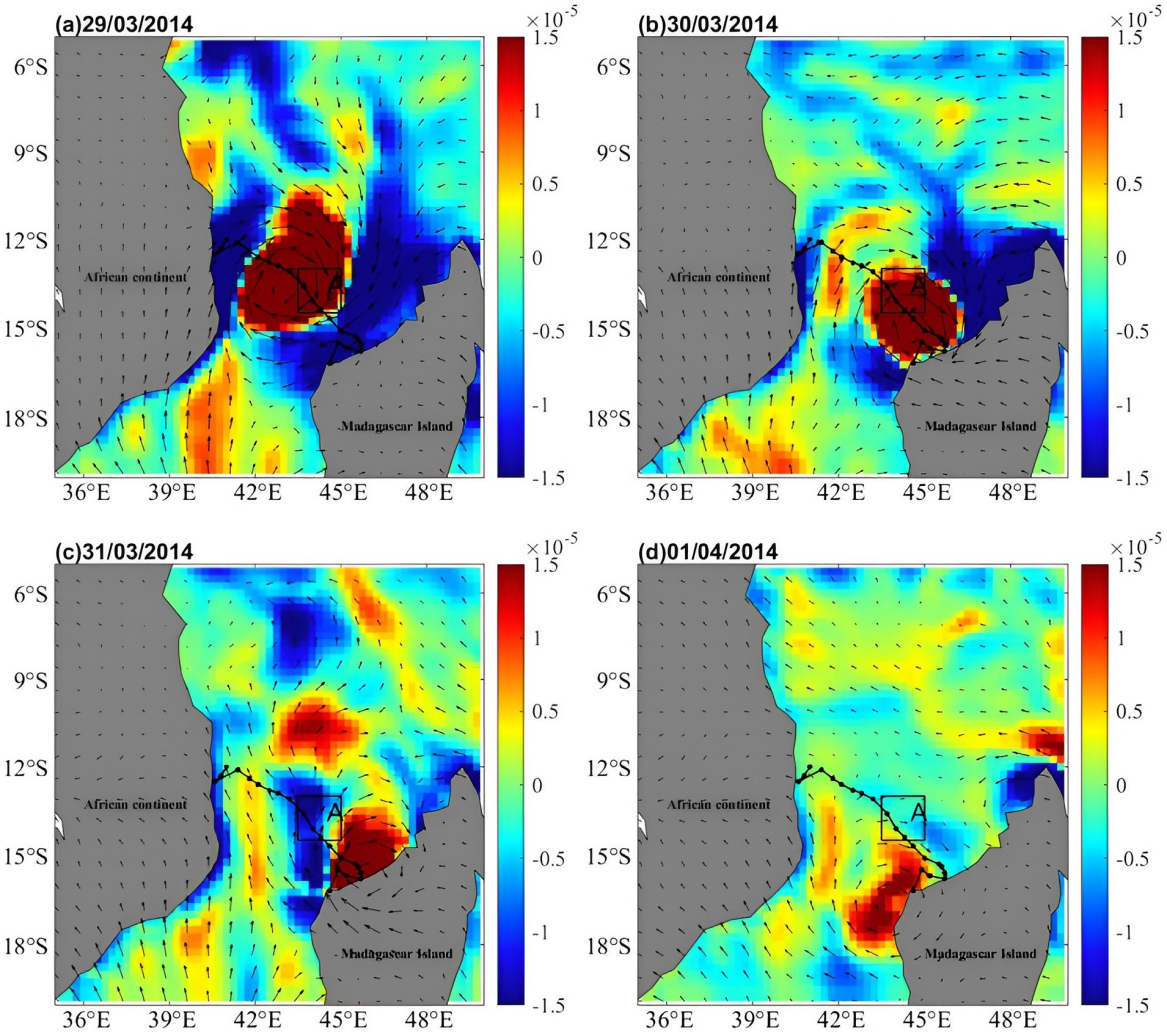
The sea surface wind velocity (arrows) and EPV (colors) during TC Hellen passage.

The EPV during the passage of Hellen was calculated from Eqs (1), (2), and (3), as shown in Fig 8. The upwelling near Box A was significantly more potent (>1.5 × 10^−5^ m∙s^-1^) on March 29 and 30 than that during the non cyclone period (Fig 8a and 8b). Upwelling occurred near the center of the TC, with more significant compensatory subsidence occurring outside the scope of upwelling, where phytoplankton first appeared in the subsurface and erupted at the surface by upwelling by Ekman pumping [60].

**Fig 9:**
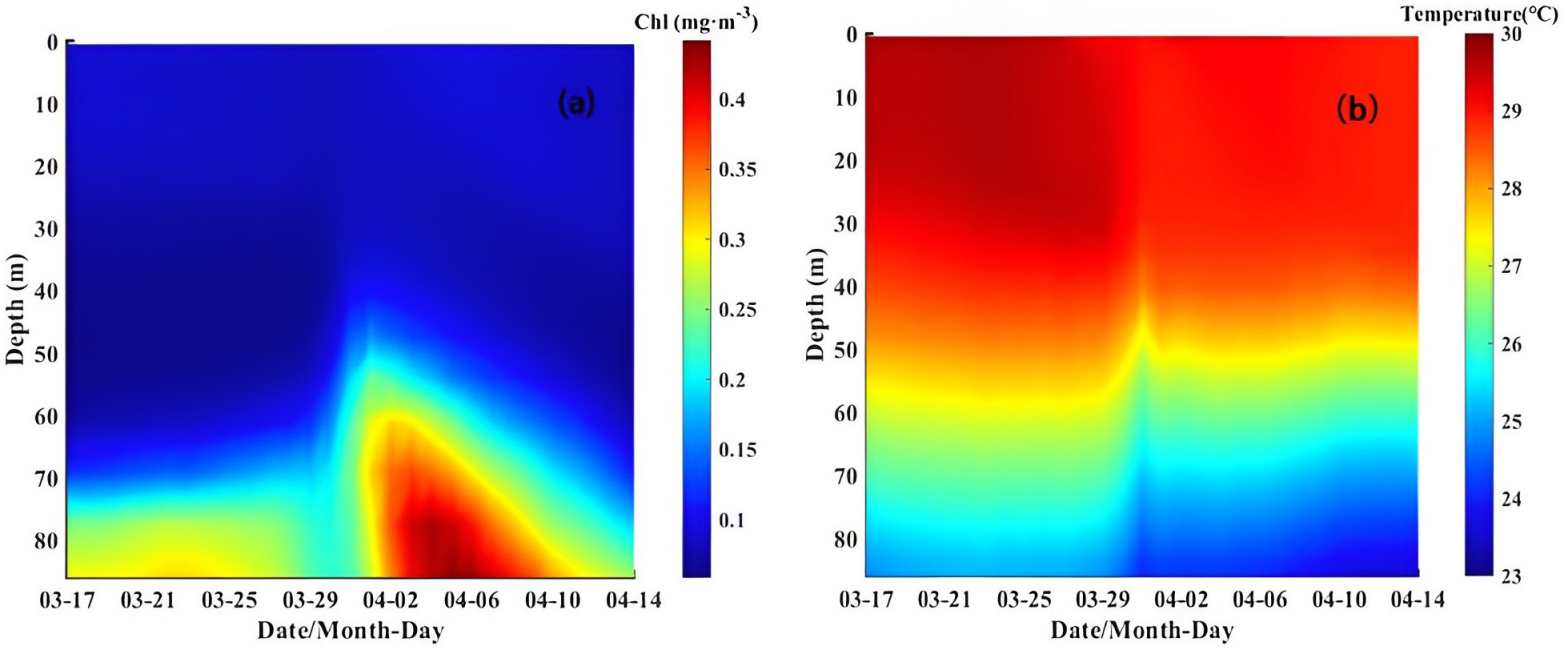
Time series of the vertical distributions of Chl-a concentration and temperature in Box A. (Note: The vertical distributions of Chl-a concentration and temperature data are from E.U. Copernicus Marine Service; https://doi.org/10.48670/moi-00019 and https://doi.org/10.48670/moi-00021).

A time series of the mean Chl-a concentration and temperature in Box A from March 17 to April 14 is shown in Fig 9. It can be found that a distinct upwelling existed near northern Mozambique (43.5°E—45°E, 14.5°S—13°S). Liu et al. (2009) suggested that the upper ocean’s dynamic response to enhanced nutrients may be significantly exacerbated after the passage of a storm [61]. Lao et al.(2022) made a relevant study on the quantification of nutrient availability in the upper ocean following TCs [12]. Cyclonic eddies have a relatively unstable thermal structure and cold water upwelling, and these factors dramatically impact seawater stratification [55, 56].

From March 30 to April 6, subsurface Chl-a invaded the surface upward by upwelling and vertical mixing, thereby strengthening the surface Chl-a bloom (Fig 9a). Meanwhile, cold water at a depth of 50 m was uplifted to the upper ocean on March 31 during the passage of the TC (Fig 9b), transporting nutrients up to the sea surface.

### Sea surface wind

**Fig 10:**
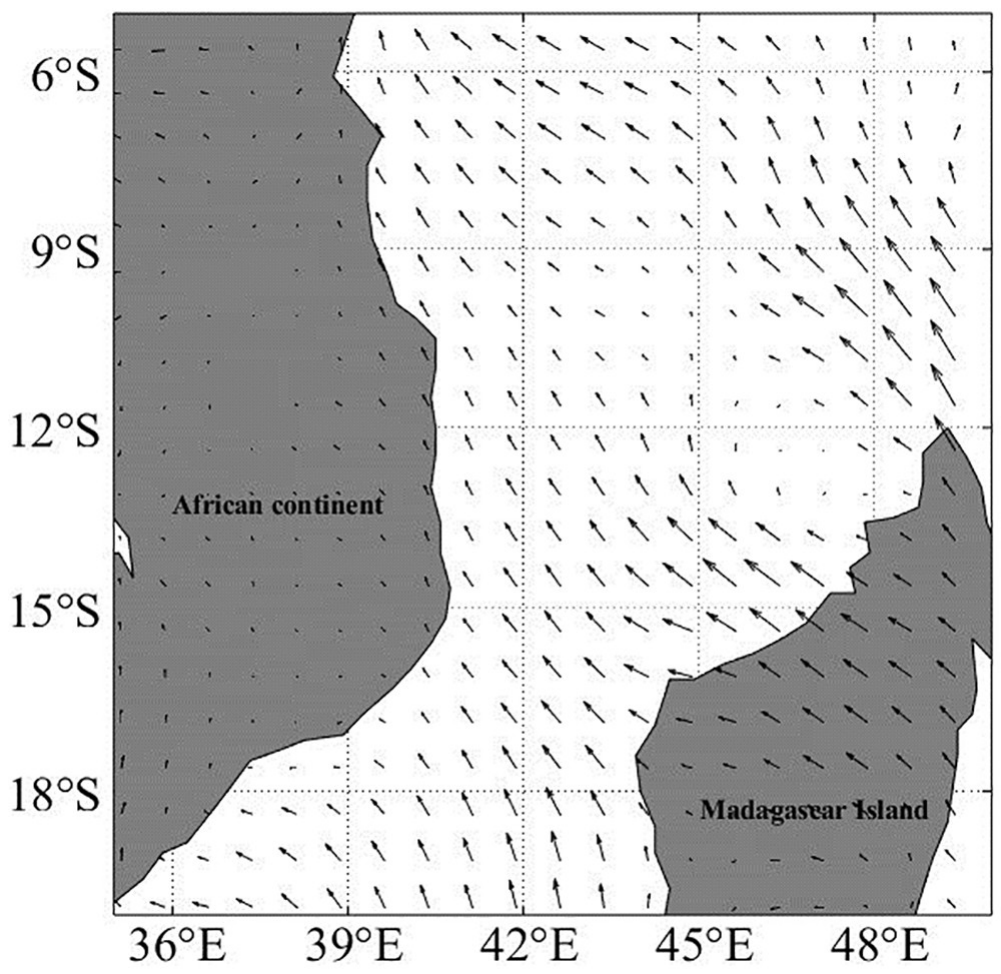
Snapshots of the sea surface wind velocity component (m∙s^-1^) showing the wind regimes: Southeast winds on April 5, 2014, corresponding with the elevated Chl-a event depicted in Fig 2.

**Fig 11:**
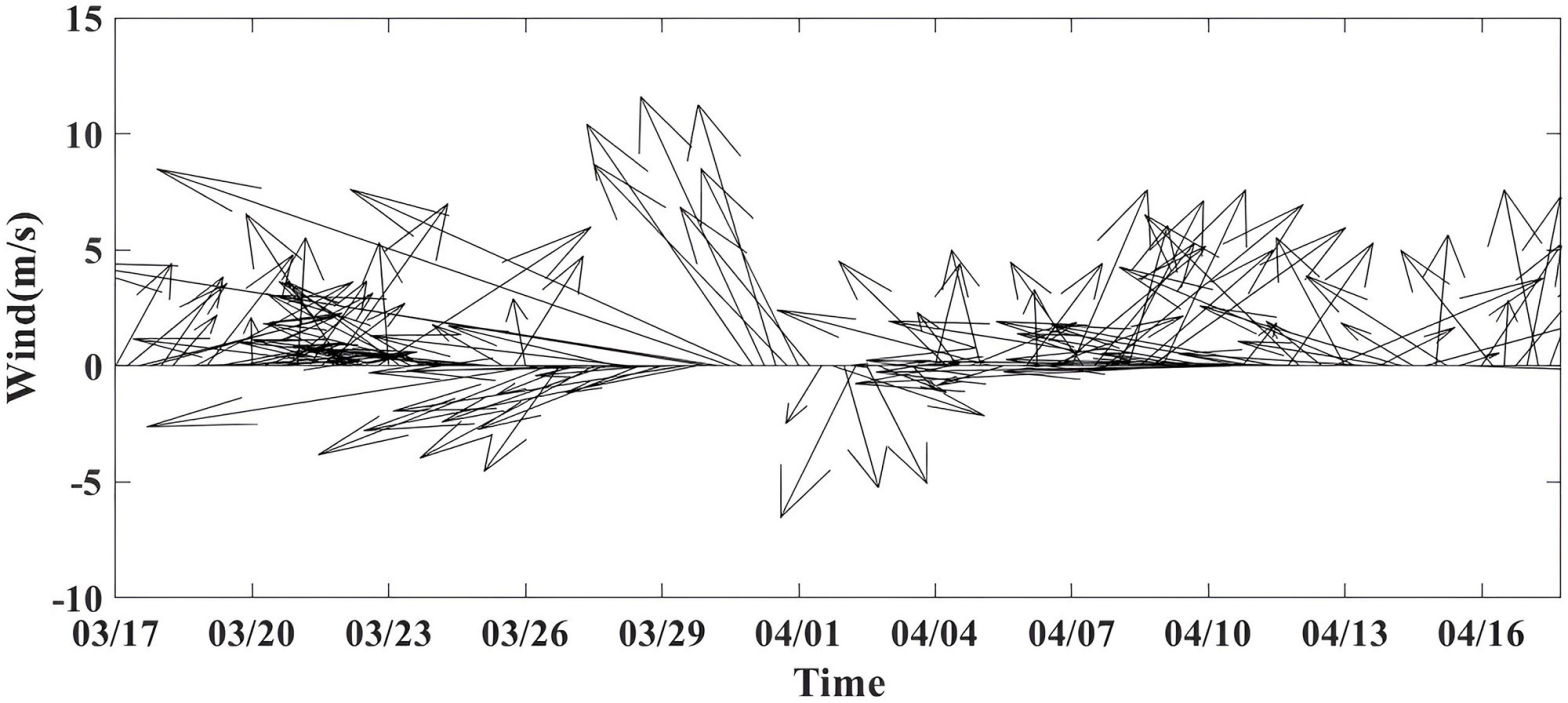
Daily alongshore blended sea surface wind velocity component (m∙s^-1^) from March 17 –April 17, 2014. Positive (negative) y-axis wind values refer to the south (north) wind.

The wind speed accumulated gradually, reaching a maximum value of 60.00 m∙s^-1^ on March 30 (Table 1 and Fig 11). After the passage of the TC, the MWS weakened to 16.3 m∙s^-1^. As shown in Fig 7a and 7b, the passage of TC Hellen induced an SST salinity response. Fig 10 is a snapshot of the Ekman-transported surface wind field on April 5, with southeast wind dominating Mozambique waters after the passage of TC Hellen. Under the influence of southeast winds, the coastal Chl-a blooms induced by upwelling were maintained for five days and transported offshore from northern Madagascar Island via ocean fronts between anticyclones and cyclones (Fig 2), coinciding with the wind-driven direction (Figs 10 and 11). The southeast wind parallel to the coast of Madagascar (~17°S) favored offshore surface Ekman transport (Fig 10), resulting in wind-induced coastal upwelling [32].

Local wind may act as a forcing factor for chlorophyll movement events [32]. The time series of UTR day-by-day onshore wind components show that the southeast wind was dominant and relatively strong (Fig 11). From March 29 to April 1, as the TC arrived at the UTR site, it induced a clockwise rotation of the winds in the nearby sea from the southeast to northwest, consistent with the direction of the TC in the Southern Hemisphere. The local wind strengthened the cyclonic eddy, which uplifted nutrients and Chl-a from the deep ocean to the upper layer.

## Conclusions

In this study, we analyzed the mechanism of the Chl-a bloom and its northwestward transport event in the northern Mozambique Channel during and after the passage of TC Hellen in 2014. The following conclusions were drawn:

1. The Chl-a concentration in Box A after the passage of the TC was significantly higher by 20-fold than that before the passage of the TC, and the TC passage lasted for five days, from April 2 to April 6. A pair of opposite polarity eddies occurred; the cyclonic cold eddy in Box A uplifted nutrients and Chl-a from the deep layer to the upper ocean with a deepened MLD and weakened barrier thickness.
2. Chl-a gradually moved from inshore to offshore along the path of TC Hellen. This was because the coastal southeast wind parallel to the coast (~17°S) of Madagascar supported offshore surface Ekman transport and wind-induced coastal upwelling.
3. TC Hellen was suppressed and weakened by the cold cyclonic eddy in Box A. On March 29, the TC passed through the cold cyclonic eddy with a significant shortage of latent heat supply; at this time, the maximum mean latent heat flux was only 137.99 W/m^2^, and the TCHP was reduced by 19.71 kJ/cm^2^ after the passage of TC Hellen.

## References

[1] Peduzzi P, Chatenoux B, Dao H, De Bono A, Herold C, Kossin J, et al. Global trends in tropical cyclone risk. Nature Climate Change. 2012;2(4):289–94. doi: 10.1038/nclimate1410

[2] Guan M, Li Q, Zhu J, Wang C, Zhou L, Huang C, et al. A method of establishing an instantaneous water level model for tide correction. Ocean Engineering. 2019;171:324–31. doi: 10.1016/j.oceaneng.2018.11.016

[3] Qiu Z, Jiao M, Jiang T, Zhou L. Dam Structure Deformation Monitoring by GB-InSAR Approach. IEEE Access. 2020;8:123287–96. doi: 10.1109/access.2020.3005343

[4] Sun P, Zhang K, Wu S, Wang R, Wan M. An investigation into real-time GPS/GLONASS single-frequency precise point positioning and its atmospheric mitigation strategies. Measurement Science and Technology. 2021;32(11). doi: 10.1088/1361-6501/ac0a0e

[5] Liu Y, Wang R, Gao J, Zhu P, Cacace F. The Impact of Different Mapping Function Models and Meteorological Parameter Calculation Methods on the Calculation Results of Single-Frequency Precise Point Positioning with Increased Tropospheric Gradient. Mathematical Problems in Engineering. 2020;2020:1–12. doi: 10.1155/2020/9730129

[6] Guan M, Cheng Y, Li Q, Wang C, Fang X, Yu J. An Effective Method for Submarine Buried Pipeline Detection via Multi-Sensor Data Fusion. IEEE Access. 2019;7:125300–9. doi: 10.1109/access.2019.2938264

[7] LÜ H, Xie J, Xu J, Chen Z, Liu T, Cai S. Force and torque exerted by internal solitary waves in background parabolic current on cylindrical tendon leg by numerical simulation. Ocean Engineering. 2016;114:250–8. doi: 10.1016/j.oceaneng.2016.01.028

[8] Mawren D, Hermes J, Reason CJC. Marine heat waves and tropical cyclones—Two devastating types of coastal hazard in South-eastern Africa. Estuarine, Coastal and Shelf Science. 2022;277:108056. doi: 10.1016/j.ecss.2022.108056

[9] Terry JP, Kim I-H, Jolivet S. Sinuosity of tropical cyclone tracks in the South West Indian Ocean: Spatio-temporal patterns and relationships with fundamental storm attributes. Applied Geography. 2013;45:29–40. doi: 10.1016/j.apgeog.2013.08.006

[10] Zhao H, Shao J, Han G, Yang D, Lv J. Influence of Typhoon Matsa on Phytoplankton Chlorophyll-a off East China. PLOS ONE. 2015;10(9):e0137863. doi: 10.1371/journal.pone.0137863 26407324 PMC4583286

[11] Lin II. Typhoon-induced phytoplankton blooms and primary productivity increase in the western North Pacific subtropical ocean. Journal of Geophysical Research: Oceans. 2012;117(C3):n/a–n/a. doi: 10.1029/2011jc007626

[12] Lao Q, Lu X, Chen F, Jin G, Chen C, Zhou X, et al. Effects of upwelling and runoff on water mass mixing and nutrient supply induced by typhoons: Insight from dual water isotopes tracing. Limnology and Oceanography. 2022;68(1):284–95. doi: 10.1002/lno.12266

[13] Araujo M, Limongi C, Servain J, Silva M, Leite FS, Veleda D, et al. Salinity-induced mixed and barrier layers in the southwestern tropical Atlantic Ocean off the northeast of Brazil. Ocean Sci. 2011;7(1):63–73. doi: 10.5194/os-7-63-2011

[14] Liu Y, Lu H, Zhang H, Cui Y, Xing X. Effects of ocean eddies on the tropical storm Roanu intensity in the Bay of Bengal. PLoS One. 2021;16(3):e0247521. Epub 20210305. doi: 10.1371/journal.pone.0247521 . 33667249 PMC7935279

[15] Li J, Zheng H, Xie L, Zheng Q, Ling Z, Li M. Response of Total Suspended Sediment and Chlorophyll-a Concentration to Late Autumn Typhoon Events in the Northwestern South China Sea. Remote Sensing [Internet]. 2021; 13(15).

[16] Chen C-TA, Liu C-T, Chuang WS, Yang YJ, Shiah F-K, Tang TY, et al. Enhanced buoyancy and hence upwelling of subsurface Kuroshio waters after a typhoon in the southern East China Sea. Journal of Marine Systems. 2003;42(1–2):65–79. doi: 10.1016/s0924-7963(03)00065-4

[17] Lu X, Zhou X, Jin G, Chen F, Zhang S, Li Z, et al. Biological Impact of Typhoon Wipha in the Coastal Area of Western Guangdong: A Comparative Field Observation Perspective. Journal of Geophysical Research: Biogeosciences. 2022;127(2). doi: 10.1029/2021jg006589

[18] Qiu D, Zhong Y, Chen Y, Tan Y, Song X, Huang L. Short-Term Phytoplankton Dynamics During Typhoon Season in and Near the Pearl River Estuary, South China Sea. Journal of Geophysical Research: Biogeosciences. 2019;124(2):274–92. doi: 10.1029/2018JG004672

[19] Lao Q, Chen F, Jin G, Lu X, Chen C, Zhou X, et al. Characteristics and Mechanisms of Typhoon‐Induced Decomposition of Organic Matter and Its Implication for Climate Change. Journal of Geophysical Research: Biogeosciences. 2023;128(6). doi: 10.1029/2023jg007518

[20] Liu S-S, Sun L, Wu Q, Yang Y-J. The responses of cyclonic and anticyclonic eddies to typhoon forcing: The vertical temperature-salinity structure changes associated with the horizontal convergence/divergence. Journal of Geophysical Research: Oceans. 2017;122(6):4974–89. doi: 10.1002/2017jc012814

[21] Li Y, Yang D, Xu L, Gao G, He Z, Cui X, et al. Three Types of Typhoon‐Induced Upwellings Enhance Coastal Algal Blooms: A Case Study. Journal of Geophysical Research: Oceans. 2022;127(5). doi: 10.1029/2022jc018448

[22] Balaji M, Chakraborty A, Mandal M. Changes in tropical cyclone activity in north Indian Ocean during satellite era (1981–2014). International Journal of Climatology. 2018;38(6):2819–37. doi: 10.1002/joc.5463

[23] Rotunno R, Emanuel KA. An Air–Sea Interaction Theory for Tropical Cyclones. Part II: Evolutionary Study Using a Nonhydrostatic Axisymmetric Numerical Model. Journal of Atmospheric Sciences. 1987;44(3):542–61. doi: 10.1175/1520-0469(1987)044&lt;0542:AAITFT&gt;2.0.CO;2

[24] Iskandar I, Sasaki H, Sasai Y, Masumoto Y, Mizuno K. A numerical investigation of eddy-induced chlorophyll bloom in the southeastern tropical Indian Ocean during Indian Ocean Dipole—2006. Ocean Dynamics. 2010;60(3):731–42. doi: 10.1007/s10236-010-0290-6

[25] Xing X, Luo S, Zhang H, Shi J, LÜ H. Phytoplankton Blooms Triggered by Anticyclonic Eddy and Cyclonic Eddy during Tropical Cyclone Nada. Tellus A: Dynamic Meteorology and Oceanography. 2023;75(1):10–23. doi: 10.16993/tellusa.147

[26] Ning J, Xu Q, Zhang H, Wang T, Fan K. Impact of Cyclonic Ocean Eddies on Upper Ocean Thermodynamic Response to Typhoon Soudelor. Remote Sensing [Internet]. 2019; 11(8).

[27] Wang G, Zhao B, Qiao F, Zhao C. Rapid intensification of Super Typhoon Haiyan: the important role of a warm-core ocean eddy. Ocean Dynamics. 2018;68(12):1649–61. doi: 10.1007/s10236-018-1217-x

[28] Bai L, Lü H, Huang H, Muhammad Imran S, Ding X, Zhang Y. Effects of Anticyclonic Eddies on the Unique Tropical Storm Deliwe (2014) in the Mozambique Channel. Journal of Marine Science and Engineering. 2023;11(1). doi: 10.3390/jmse11010129

[29] Chacko N, Jayaram C. Response of the Bay of Bengal to super cyclone Amphan examined using synergistic satellite and in-situ observations. Oceanologia. 2022;64(1):131–44. doi: 10.1016/j.oceano.2021.09.006

[30] Xia C, LÜ H, Shen H, Muhammad I S, Ding X. What happened around an inverted V-shaped track turning of the tropical cyclone Madi? Journal of Sea Research. 2023;191. doi: 10.1016/j.seares.2022.102324

[31] Barlow R, Lamont T, Morris T, Sessions H, van den Berg M. Adaptation of phytoplankton communities to mesoscale eddies in the Mozambique Channel. Deep Sea Research Part II: Topical Studies in Oceanography. 2014;100:106–18. doi: 10.1016/j.dsr2.2013.10.020

[32] Malauene BS, Shillington FA, Roberts MJ, Moloney CL. Cool, elevated chlorophyll-a waters off northern Mozambique. Deep Sea Research Part II: Topical Studies in Oceanography. 2014;100:68–78. doi: 10.1016/j.dsr2.2013.10.017

[33] Schouten MW, de Ruijter WPM, van Leeuwen PJ, Ridderinkhof H. Eddies and variability in the Mozambique Channel. Deep Sea Research Part II: Topical Studies in Oceanography. 2003;50(12):1987–2003. doi: 10.1016/S0967-0645(03)00042-0

[34] Cai J, Zhang Y, Li Y, Liang XS, Jiang T. Analyzing the Characteristics of Soil Moisture Using GLDAS Data: A Case Study in Eastern China. Applied Sciences [Internet]. 2017; 7(6).

[35] Mawren D, Blamey R, Hermes J, Reason CJC. On the importance of the Mozambique Channel for the climate of southeastern Africa. Climate Dynamics. 2023;60(1):279–99. doi: 10.1007/s00382-022-06334-w

[36] Lin II, Wu C-C, Emanuel KA, Lee IH, Wu C-R, Pun I-F. The Interaction of Supertyphoon Maemi (2003) with a Warm Ocean Eddy. Monthly Weather Review. 2005;133(9):2635–49. doi: 10.1175/MWR3005.1

[37] Gaube P, Chelton DB, Strutton PG, Behrenfeld MJ. Satellite observations of chlorophyll, phytoplankton biomass, and Ekman pumping in nonlinear mesoscale eddies. Journal of Geophysical Research: Oceans. 2013;118(12):6349–70. doi: 10.1002/2013JC009027

[38] Lu H, Zhao X, Sun J, Zha G, Xi J, Cai S. A case study of a phytoplankton bloom triggered by a tropical cyclone and cyclonic eddies. PLoS One. 2020;15(4):e0230394. Epub 20200410. doi: 10.1371/journal.pone.0230394 . 32275722 PMC7147795

[39] Wang T, Zhang S, Chen F, Ma Y, Jiang C, Yu J. Influence of sequential tropical cyclones on phytoplankton blooms in the northwestern South China Sea. Journal of Oceanology and Limnology. 2021;39(1):14–25. doi: 10.1007/s00343-020-9266-7

[40] de Boyer Montégut C. Mixed layer depth over the global ocean: An examination of profile data and a profile-based climatology. Journal of Geophysical Research. 2004;109(C12). doi: 10.1029/2004jc002378

[41] Park K-A, Lee E-Y, Chang E, Hong S. Spatial and temporal variability of sea surface temperature and warming trends in the Yellow Sea. Journal of Marine Systems. 2015;143:24–38. doi: 10.1016/j.jmarsys.2014.10.013

[42] Sadhuram Y, Rao BP, Rao DP, Shastri PNM, Subrahmanyam MV. Seasonal Variability of Cyclone Heat Potential in the Bay of Bengal. Natural Hazards. 2004;32(2):191–209. doi: 10.1023/B:NHAZ.0000031313.43492.a8

[43] Vissa NK, Satyanarayana ANV, Prasad Kumar B. Response of Upper Ocean during passage of MALA cyclone utilizing ARGO data. International Journal of Applied Earth Observation and Geoinformation. 2012;14(1):149–59. doi: 10.1016/j.jag.2011.08.015

[44] Jangir B, Swain D, Ghose SK. Influence of eddies and tropical cyclone heat potential on intensity changes of tropical cyclones in the North Indian Ocean. Advances in Space Research. 2021;68(2):773–86. doi: 10.1016/j.asr.2020.01.011

[45] Xia C, Ge X, LÜ H, Zhang H, Xing X, Cui Y. A phytoplankton bloom with a cyclonic eddy enhanced by the tropical cyclone Phethai in eastern Sir Lanka. Regional Studies in Marine Science. 2022;51:102217. doi: 10.1016/j.rsma.2022.102217

[46] Kumar BP, D’Asaro E, Suresh kumar N, Ravichandran M. Widespread cooling of the Bay of Bengal by tropical storm Roanu. Deep Sea Research Part II: Topical Studies in Oceanography. 2019;168:104652. doi: 10.1016/j.dsr2.2019.104652

[47] Xu H, Tang D, Liu Y, Li Y. Dissolved oxygen responses to tropical cyclones "Wind Pump" on pre-existing cyclonic and anticyclonic eddies in the Bay of Bengal. Marine Pollution Bulletin. 2019;146:838–47. doi: 10.1016/j.marpolbul.2019.07.019 31426226

[48] Walker ND, Leben RR, Balasubramanian S. Hurricane-forced upwelling and chlorophyllaenhancement within cold-core cyclones in the Gulf of Mexico. Geophysical Research Letters. 2005;32(18):n/a–n/a. doi: 10.1029/2005gl023716

[49] Zheng Z, Ho CR, Kuo N-J. Importance of pre‐existing oceanic conditions to upper ocean response induced by Super Typhoon Hai‐Tang. Geophysical Research Letters. 2008;35.

[50] Anandh TS, Das BK, Kuttippurath J, Chakraborty A. A coupled model analyses on the interaction between oceanic eddies and tropical cyclones over the Bay of Bengal. Ocean Dynamics. 2020;70(3):327–37. doi: 10.1007/s10236-019-01330-x

[51] He Q, Zhan H, Cai S. Anticyclonic Eddies Enhance the Winter Barrier Layer and Surface Cooling in the Bay of Bengal. Journal of Geophysical Research: Oceans. 2020;125(10). doi: 10.1029/2020jc016524

[52] Ginis I, Yablonsky RM. Impact of a Warm Ocean Eddy’s Circulation on Hurricane-Induced Sea Surface Cooling with Implications for Hurricane Intensity. Monthly Weather Review. 2012;141(3):997–1021. doi: 10.1175/mwr-d-12-00248.1

[53] Ma Z, Fei J, Liu L, Huang X, Li Y. An Investigation of the Influences of Mesoscale Ocean Eddies on Tropical Cyclone Intensities. Monthly Weather Review. 2017;145(4):1181–201. doi: 10.1175/MWR-D-16-0253.1

[54] Jaimes B, Shay LK, Halliwell GR. The Response of Quasigeostrophic Oceanic Vortices to Tropical Cyclone Forcing. Journal of Physical Oceanography. 2011;41(10):1965–85. doi: 10.1175/JPO-D-11-06.1

[55] Ma Z, Fei J, Huang X, Cheng X. Modulating Effects of Mesoscale Oceanic Eddies on Sea Surface Temperature Response to Tropical Cyclones Over the Western North Pacific. Journal of Geophysical Research: Atmospheres. 2018;123(1):367–79. doi: 10.1002/2017jd027806

[56] Walker ND, Leben RR, Pilley CT, Shannon M, Herndon DC, Pun I-F, et al. Slow translation speed causes rapid collapse of northeast Pacific Hurricane Kenneth over cold core eddy. Geophysical Research Letters. 2014;41(21):7595–601. doi: 10.1002/2014gl061584

[57] Roy Chowdhury R, Prasanna Kumar S, Narvekar J, Chakraborty A. Back‐to‐Back Occurrence of Tropical Cyclones in the Arabian Sea During October–November 2015: Causes and Responses. Journal of Geophysical Research: Oceans. 2020;125(6). doi: 10.1029/2019jc015836

[58] Li J, Yang Y, Wang G, Cheng H, Sun L. Enhanced Oceanic Environmental Responses and Feedbacks to Super Typhoon Nida (2009) during the Sudden-Turning Stage. Remote Sensing [Internet]. 2021; 13(14).

[59] Sun L, Li Y-X, Yang Y-J, Wu Q, Chen X-T, Li Q-Y, et al. Effects of super typhoons on cyclonic ocean eddies in the western North Pacific: A satellite data-based evaluation between 2000 and 2008. Journal of Geophysical Research: Oceans. 2014;119(9):5585–98. doi: 10.1002/2013jc009575

[60] Jaimes B, Shay LK. Enhanced Wind-Driven Downwelling Flow in Warm Oceanic Eddy Features during the Intensification of Tropical Cyclone Isaac (2012): Observations and Theory. Journal of Physical Oceanography. 2015;45(6):1667–89. doi: 10.1175/JPO-D-14-0176.1

[61] Liu X, Wang M, Shi W. A study of a Hurricane Katrina–induced phytoplankton bloom using satellite observations and model simulations. Journal of Geophysical Research. 2009;114(C3). doi: 10.1029/2008jc004934

